# Recurrent Left Atrial Myxoma: The Significance of Active Surveillance

**DOI:** 10.7759/cureus.33990

**Published:** 2023-01-20

**Authors:** Oluwaremilekun Tolu-Akinnawo, Rabira R Dufera, Nagendra Ramanna

**Affiliations:** 1 Internal Medicine, Meharry Medical College, Nashville, USA; 2 Cardiology, Nashville General Hospital, Nashville, USA

**Keywords:** active, surveillance, transthoracic echocardiogram, left atrial mass, atrial myxoma

## Abstract

Cardiac myxoma recurrence is uncommon following surgical resection. Recurrence is about 2-3% in familial cases; however, recurrence is uncommon in sporadic cases. Most of the recurrences will occur during the first three to four years. Ten percent of myxomas are of the inherited autosomal dominant disorder called Carney's complex, while the rest appear sporadic. We are reporting a nonfamilial case of atrial myxoma, recurring rapidly seven years after resection of the initial left atrial myxoma with a pathologically proven clear margin and no malignant transformation.

Cardiac neoplasms are rare and occur less commonly than metastatic disease of the heart. Congestive heart failure symptoms and thromboembolism account for nearly half of the presenting signs and symptoms. The initial presentation of our case was an embolic phenomenon, presenting with a stroke. The patient subsequently underwent resection of the mass, with pathology confirming the complete excision of the myxoma with a clear margin and no evidence of malignant transformation. Our patient was closely followed up in the clinic on annual transthoracic echocardiography surveillance, with a recurrence noted on surveillance echocardiography in 2021 (seven years after initial diagnosis) despite the patient being asymptomatic. This case illustrates transthoracic echocardiography as the mainstay of detection of recurrent left atrial myxoma; however, it also asks the question of how often patients need to be screened for recurrence of left atrial myxoma and for how long they need to have surveillance echocardiography. Clinical presentation and transesophageal echocardiographic views are extremely helpful in sharpening the accuracy of the diagnosis.

## Introduction

Atrial myxoma is the most common primary cardiac neoplasm, with an estimated incidence of about 0.5-1.0 per million cases per year [1]. Most cases of atrial myxoma are sporadic and left-sided. They are mainly considered benign diseases; however, they can become life-threatening when they interfere with heart function. There is also a good long-term survival rate after surgical intervention [1]. Although rare, multiple recurrences of cardiac myxoma are a potential concern due to its biologically invasive characteristics [2].

## Case presentation

**Figure 1:**
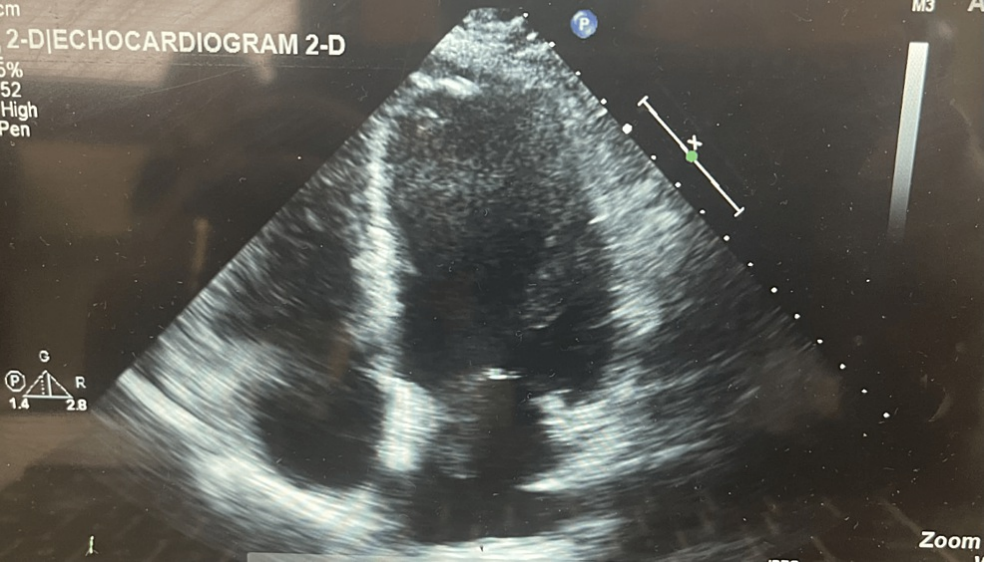
Two-dimensional ECHO done significant for recurrent left atrial myxoma

A 60-year-old female patient with a prior history of CVA, diagnosed in 2014 and on further workup, was found to have a mass in the left atrium, which was subsequently diagnosed as a left atrial myxoma on histology following a resection (April 27, 2014). The patient at that time presented for regular follow-up with complaints of fatigue and exertional dyspnea. Transthoracic echocardiography during this time showed a mobile mass attached to the inter-atrial septum prolapsing into the LV through the mitral valve in diastole. The patient subsequently underwent resection of the mass, with pathology confirming the complete excision of the myxoma with a clear margin and no evidence of malignant transformation. The patient has been closely following up with the cardiology clinic with annual transthoracic echocardiography per the Dictionary of Occupational Titles requirement for a return to work for bus drivers. In July 2021, the patient, however, presented to the cardiology clinic for regular follow-up without any new symptoms. Transthoracic echocardiography was significant for the new finding of a 1.6 × 1.5 cm echogenic mobile mass attached to the proximal portion of the left side of the interatrial septum, consistent with a left atrial myxoma at the previous area of the stack (Figure 1).

This was absent in the transthoracic echocardiography done a year prior, which indicates a rapid development of left atrial myxoma recurrence. The patient subsequently had a resection of the recurrent left atrial myxoma done on July 2021, and the pathology report from the second surgery did not show malignancy transformation. This case illustrates a recurrence of left atrial myxoma after seven years of initial diagnosis; however, it also illustrates the sudden and rapid growth within a year, as the transthoracic echocardiography done in 2020 was negative for left atrial myxoma. This begs the question; how often do we screen asymptomatic patients for left atrial myxoma recurrence?

## Discussion

Atrial myxoma is the most common type of benign cardiac tumor, responsible for about 80% of primary cardiac tumors [3]. Crafoord first discovered atrial myxoma in 1954; however, the first case of recurrence four years after the removal of the first tumor was reported by Gerbode in 1967 [3]. However, recurrent atrial myxoma still remains infrequent, with an estimated recurrence rate of 2-3% [3]. The most common site of atrial myxoma is the left atrium, with approximately 80% of cases found in this site. There is also a higher prevalence rate in females than males, with most patients in the 3rd-6th decades of life [4]. However, tumor recurrence is more common in men [5].

Symptomatic patients are usually present based on the tumor size, site of the tumor, degree of obstruction, and effect of the tumor on the cardiac valves [6]. Most patients present with signs of obstructive ventricular filling and pulmonary edema, such as dyspnea, orthopnea, and paroxysmal nocturnal dyspnea. Less commonly, atrial myxoma could present as chest pain, syncope, stroke, or palpitation [6]. Symptoms such as the production and release of interleukin, which is common with neoplastic cells, are responsible for most of the constitutional symptoms such as fatigue, weight loss, fever, and arthralgia. Ischemic stroke is the most common neurologic manifestation of cardiac myxoma, with an estimated 0.5% of ischemic stroke cases attributed to myxomatous embolization, just as in the case of our patient [7]. Patients can also be asymptomatic, as in the case of our patient.

Although the pathophysiology of recurrence is still poorly understood, four different mechanisms have been postulated. These mechanisms are "inadequate resection, totipotent multicentricity, inheritance (family type), and metastatic recurrence" [5]. In most sporadic myxoma cases, incomplete resection of the tumor is responsible for recurrence [1].

The presence of concurrent lesions in the heart and clinical features such as hyperpigmentation, endocrine disease, or benign extracardiac connective tissue suggestive of syndromic association (Carney complex) should serve as a pointer to a familial transmission [5]. Familial transmission has been linked with causative mutations [8]. For the familiar pathology, the recurrence rate can be as high as 12% [1].

Recurrence of atrial myxoma after surgical resection is rare, usually less than 5% of cases. In the case of our patient, the first recurrence occurred seven years after the initial complete resection. This case illustrates a recurrence after surgical resection, which is extremely rare, with only 10 cases of multiple recurrences reported in the literature in the past 30 years [9]. This case also shows the significance of regular echocardiography after surgical resections to identify such recurrences quickly. However, this case is also unique due to the rapid growth of the recurrent left atrial myxoma within a year despite the patient being asymptomatic (transthoracic echocardiography done between 2015 and 2020 were all negative for left atrial myxoma). This begs the question: how often do patients with left atrial myxoma need to be screened with a transthoracic echocardiogram after resecting the mass? Also, for how long do they need to be screened or followed up? This case also demonstrates the need for genetic analysis of patients with recurrent cardiac myxomas to investigate the nature of these tumors.

## Conclusions

Recurrence of the most common cardiac, primary benign tumor-atrial myxoma, is rare, especially after multiple surgeries. Sporadic cases are less likely to be recurrent than familial cases. Our patient not only presented with a sporadic cause but also recurred seven years after resection with a pathologically proven clear margin and no malignant transformation. Our case also illustrates the risk of rapid progression despite previous negative screenings. The risk of recurrence, despite surgical resection, and the potential for worsening outcomes demonstrate the need for regular echocardiography surveillance post-resection. However, the question is: how often do we need patients to have surveillance echocardiography, and for how long?

Further studies will help address these questions. It is important to detect recurrences early, especially in patients with a familial predisposition. However, additional studies are also needed to understand the reliable predictors of atrial myxoma recurrence better and more accurately.
